# Test for Aviators

**Published:** 1919-01

**Authors:** 


*Test for Aviators.* By C. M. Robertson, Chicago. *Journal A. M. A.* Sept. 7, 1918.

A new test and method of classification for labyrinth, muscle-tone and blood pressure findings is described by the author in a preliminary report on the examination of aviators.

To be an aviator a man must be physically qualified both as to eyesight and equilibrium tests, and past pointing and falling. For very low altitude maneuvering, when the density and conditions are much the same as those on the earth's surface, the man does not have to be so perfect, but this does not hold true for the aviator who ascends more than a thousand feet. If they could ascend or descend at only a moderate rate of speed, they would not have to be chosen with so much care. The aviator must change his altitude suddenly, and to a very great amount in many cases, so that he is subjected to violent changes of temperature and air pressure, sometimes within a few seconds. Motor manufacturers have had to recognize this in the equipment of carbureters, for it is necessary to have a regulating device to feed into the gasoline mixture at 15 000 feet three times the amount of oxygen that is required at the earth's surface. The temperature at 25 000 feet is far below zero (F.), while the sun's rays register nearly 140 F. As we ascend, the content of the air is not changed, but the oxygen tension is diminished, at 16 000 feet it being only 25 per cent. of the tension at the earth's surface. Aeroplanes are now being supplied with a device by which oxygen is fed to the engine and to the aviator automatically as the altitude becomes greater, and which diminishes the oxygen supplied as the altitude becomes less. At the present time, too, a machine must be able to leave the ground almost immediately and be able to climb at the rate of 1 000 feet a minute. The speed of the present machines in the German army, we are told, is not less than 140 miles an hour, while their scouting craft do as much as 200 miles an hour. It is not enough to have merely a sound body and a sound mind. Some people thus supplied are liable to have dizzy spells, seasickness and fainting. Aviators are required not merely to fly but to perform all sorts of evolutions and experience violent changes of atmospheric pressure, temperature, etc. In doing these, and in judging of the ability of an applicant for this service, we should test him in some ways that resemble as nearly as possible an actual flight with all its maneuvers. Robertson has devised a cabinet which he believes meets all the requirements. Reading of the accidents and accounts of combats, he felt there must be a reason why so many fell from causes other than damage received from an opponent. Faults of the machine may be due to exhaustion of gasoline, fouling of the motor, or injury in combat. The operator may become entirely exhausted, or so dizzy as to lose control, or may have lost his motor power from great atmospheric changes, or hemorrhage into the labyrinth, or heart failure. He describes the method of application of the test and the apparatus. Extensive tables accompany the paper, and they show that high or low blood pressure strongly affects the capacity of the aviator, and the results of the tests, as showing the fitness of the man for the work, are pointed out. The apparatus is illustrated.

